# Adverse effects of endometriosis on pregnancy: a case-control study

**DOI:** 10.1186/s12884-019-2514-1

**Published:** 2019-10-22

**Authors:** Mayo Miura, Takafumi Ushida, Kenji Imai, Jingwen Wang, Yoshinori Moriyama, Tomoko Nakano-Kobayashi, Satoko Osuka, Fumitaka Kikkawa, Tomomi Kotani

**Affiliations:** 1 Department of Obstetrics and Gynecology, Nagoya University Graduate School of Medicine, 65 Tsurumai-cho, Showa-ku, Nagoya, 466-8550 Japan; 2 Laboratory of Bell Research Center-Department of Obstetrics and Gynecology collaborative research, Nagoya University Graduate School of Medicine, 65 Tsurumai-cho, Showa-ku, Nagoya, 466-8550 Japan; 3 Center for Maternal-Neonatal Care, Nagoya University Hospital, 65 Tsurumai-cho, Showa-ku, Nagoya, 466-8560 Japan; 4 Department of Maternal and Perinatal Medicine, Nagoya University Hospital, 65 Tsurumai-cho, Showa-ku, Nagoya, 466-8560 Japan

**Keywords:** Endometriosis, Pregnancy complications, Neonatal outcomes, Placenta previa

## Abstract

**Background:**

Endometriosis is a common disease occurring in 1–2% of all women of reproductive age. Although there is increasing evidence on the association between endometriosis and adverse perinatal outcomes, little is known about the effect of pre-pregnancy treatments for endometriosis on subsequent perinatal outcomes. Thus, this study aimed to evaluate maternal and neonatal outcomes in pregnant women with endometriosis and to investigate whether pre-pregnancy surgical treatment would affect these outcomes.

**Methods:**

This case-control study included 2769 patients who gave birth at Nagoya University Hospital located in Japan between 2010 and 2017. Maternal and neonatal outcomes were compared between the endometriosis group (*n* = 80) and the control group (*n* = 2689). The endometriosis group was further divided into two groups: patients with a history of surgical treatment such as cystectomy for ovarian endometriosis, ablation or excision of endometriotic implants, or adhesiolysis (surgical treatment group, *n* = 49) and those treated with only medications or without any treatment (non-surgical treatment group, *n* = 31).

**Results:**

In the univariate analysis, placenta previa and postpartum hemorrhage were significantly increased in the endometriosis group compared to the control group (12.5% vs. 4.1%, *p* <  0.01 and 27.5% vs. 18.2%, *p* = 0.04, respectively). In the multivariate analysis, endometriosis significantly increased the odds ratio (OR) for placenta previa (adjusted OR, 3.19; 95% confidence interval [CI], 1.56–6.50, *p* <  0.01) but not for postpartum hemorrhage (adjusted OR, 1.14; 95% CI, 0.66–1.98, *p* = 0.64). Other maternal and neonatal outcomes were similar between the two groups. In patients with endometriosis, patients in the surgical treatment group were significantly associated with an increased risk of placenta previa (OR. 4.62; 95% CI, 2.11–10.10, *p* <  0.01); however, patients in the non-surgical treatment group were not associated with a high risk (OR, 1.63; 95% CI, 0.19–6.59, *p* = 0.36). Additionally, other maternal and neonatal outcomes were similar between the two groups.

**Conclusion:**

Women who have had surgical treatment for their endometriosis appear to have a higher risk for placenta previa. This may be due to the more severe stage of endometriosis often found in these patients. However, clinicians should be alert to this potential increased risk and manage these patients accordingly.

## Background

Endometriosis is a common chronic gynecological disorder defined as the presence of endometrial-like glands and stroma outside the uterus such as in the ovary and peritoneum [1]. The prevalence of endometriosis is 1–2% of all women of reproductive age and 3–11% of infertile women [2–7]. Common symptoms of endometriosis are infertility, dysmenorrhea, chronic pelvic pain, dyspareunia, and painful defecation [8, 9].

Surgical procedures such as laparoscopic cystectomy, excision, ablation, and adhesiolysis are considered effective treatments to reduce chronic pain when conservative treatments have insufficient efficacy [10]. However, a 5-year recurrence rate of 40–50% after surgery and the possible harmful effect on ovarian reserve is often problematic [2, 11].

In the past 10 years, increasing evidence on the association between endometriosis and increased risk of pregnancy complications such as placenta previa, preterm birth, hypertensive disorders of pregnancy (HDP), small for gestational age (SGA), placental abruption, and postpartum hemorrhage (PPH) is observed [12–15]. Presently, the effects of endometriosis on perinatal outcomes have remained controversial [16, 17]. Additionally, whether pre-pregnancy treatments such as surgical treatment or hormone therapies for infertility, ovarian endometriosis, and chronic pain due to endometriosis improve perinatal outcomes in subsequent pregnancies remains uncertain and is an important clinical question. Thus, this study aimed to clarify the effects of endometriosis on maternal and neonatal outcomes and to investigate whether pre-pregnancy surgical treatment for endometriosis affects these outcomes.

## Methods

This case-control study was conducted to compare maternal and neonatal outcomes between women with and without endometriosis who gave birth at Nagoya University Hospital between January 2010 and December 2017. Nagoya University Hospital is located in a metropolitan area in Japan and specializes in high-risk pregnancies. This study was approved by the Institutional Review Board of Nagoya University (approval number: 2015–0415). The exclusion criteria for this study were as follows: less than 22 weeks of gestation at birth, fetal malformations, multiple pregnancies, and incomplete medical records. This study included 80 patients diagnosed with endometriosis (endometriosis group) and 2689 patients without endometriosis (control group). All data on maternal and neonatal characteristics were obtained by a manual search of the electronic medical record system. In this study, diagnosis of endometriosis was based on laparoscopy with histological confirmation (*n* = 49), ultrasound or magnetic resonance imaging (MRI) with ovarian endometriosis (*n* = 27), and presence of symptoms (*n* = 4). Patients who were diagnosed with endometriosis before pregnancy or who were diagnosed with ovarian endometriosis in the first trimester were included in the endometriosis group. The endometriosis group was further divided into two groups: patients with a history of surgical treatments such as cystectomy for ovarian endometriosis, ablation or excision of endometriotic implants, and adhesiolysis (surgical treatment group, *n* = 49) and those treated with only medications or without any treatment (non-surgical treatment group, *n* = 31). In the non-surgical treatment group, 26 patients were diagnosed with presence of symptoms before pregnancy (*n* = 4) and ovarian endometriosis before pregnancy by ultrasound or MRI (*n* = 22), and 5 patients were diagnosed with ovarian endometriosis in the first trimester by ultrasound. Detailed information on treatments for endometriosis before pregnancy such as previous history of surgical treatment and hormone therapies (oral contraceptives, progestogens, and gonadotropin-releasing hormone agonists [GnRHa]) was obtained from the patients’ medical records.

Maternal characteristics included the following: maternal age, parity, body mass index (BMI) before pregnancy, chronic hypertension (HT) and diabetes mellitus (DM) before pregnancy, and assisted reproductive technology (ART). ART included in vitro fertilization and intracytoplasmic sperm injection, and not artificial insemination with husband’s semen.

The maternal outcomes included gestational age, delivery mode, blood loss at delivery, and pregnancy complications such as preterm birth (< 37 gestational weeks), HDP, gestational diabetes mellitus (GDM), PPH, placental abruption, and placenta previa. Neonatal characteristics included birth weight, height, SGA, sex, Apgar score at 1 and 5 min, umbilical artery pH, and neonatal intensive care unit (NICU) admission. SGA was defined as birth weight and height less than the 10th percentile for gestational age based on the Japanese gender-specific neonatal anthropometric chart, 2000 [18].

Previous papers have reported that the 90th percentile of blood loss including amniotic fluid within 24 h after delivery was 800 mL in a singleton vaginal delivery and 1500 mL in a singleton cesarean section [19, 20]. In this study, PPH was defined as greater than 800 mL of estimated blood loss including amniotic fluid in a singleton vaginal delivery and greater than 1500 mL of estimated blood loss including amniotic fluid in a singleton cesarean section.

All data were analyzed using the R software version 3.5.0 (www.r-project.org) and EZR (Saitama Medical Center, Jichi Medical University, Saitama, Japan) [21]. For the analysis, maternal and neonatal characteristics were compared using the chi-squared test for the categorical variables and the unpaired t-test or Mann-Whitney U tests for the continuous variables according to normal or non-normal distributions. To determine the association between endometriosis and placenta previa or PPH, multiple logistic regression analyses were performed. The respective adjusted odds ratios (aORs) and 95% confidence intervals (CIs) were calculated after the adjustment of confounding factors including pre-pregnancy BMI, maternal age at delivery, ART, and parity for placenta previa, including pre-pregnancy BMI, maternal age at delivery, ART, parity, placenta previa, and macrosomia for PPH. *P* <  0.05 was considered statistically significant.

## Results

Table 1 shows the maternal characteristics in the endometriosis and control groups. The patients in the endometriosis group were significantly older (34.2 ± 4.6 vs. 32.9 ± 5.2 years old, *p* = 0.03) and more likely to be primipara (83.8% vs. 54.7%, *p* <  0.01) and conceive using ART (28.7% vs. 12.8%, *p* <  0.01) than the control group. No significant differences were observed in pre-pregnancy BMI, HT, and DM before pregnancy between the two groups (Table 1).

**Table 1:** Maternal Characteristics in the Endometriosis and Control groups

Maternal characteristics	Endometriosis (*N* = 80)	Control (*N* = 2689)	*p*-value
Age (years)	34.2 ± 4.6	32.9 ± 5.2	0.03
Primipara	67 (83.8)	1471 (54.7)	< 0.01
Pre-Pregnancy BMI (kg/m^2^)	20.6 ± 2.6	21.2 ± 3.6	0.33
Pre-pregnancy HT	0 (0)	30 (1.1)	0.69
Pre-pregnancy DM	0 (0)	45 (1.7)	0.47
ART	23 (28.7)	343 (12.8)	< 0.01

Data are presented as mean ± standard deviation or *n* (%). *BMI* Body Mass Index, *HT* Hypertension, *DM* Diabetes Mellitus, *ART* Assisted Reproductive Technology

Maternal outcomes are shown in Table 2. There were significant differences in blood loss between the endometriosis and control groups (752 ± 688 mL vs. 560 ± 360 mL at normal vaginal delivery, *p* = 0.04, and 1346 ± 675 mL vs. 1099 ± 692 mL at scheduled cesarean section, *p* = 0.01). There were no significant differences in gestational age, delivery mode, blood loss on instrumental delivery, and emergency cesarean section between the two groups. Regarding pregnancy complications, the risk of placenta previa and PPH in the endometriosis group was significantly higher compared to the control group (12.5% vs. 4.1%, *p* < 0.01 and 27.5% vs. 18.2%, *p* = 0.04, respectively). A significant difference was not observed in the occurrences of preterm birth (< 37 weeks), HDP, GDM, and placental abruption between the two groups.

**Table 2:** Maternal Outcomes in the Endometriosis and Control groups

Maternal outcomes	Endometriosis (*N* = 80)	Control (*N* = 2689)	*p*-value
Gestational age (weeks)	38.3 ± 2.1	38.4 ± 2.4	0.34
Delivery mode			
Normal Vaginal	30 (37.5)	1245 (46.3)	
Instrumental	7 (8.8)	267 (9.9)	
Scheduled Cesarean Section	30 (37.5)	681 (25.3)	
Emergency Cesarean Section	13 (16.2)	496 (18.4)	0.11
Blood Loss (mL)			
Normal Vaginal	752 ± 688	560 ± 360	0.04
Instrumental	1000 ± 421	787 ± 557	0.10
Scheduled Cesarean Section	1346 ± 675	1099 ± 692	0.01
Emergency Cesarean Section	852 ± 314	944 ± 566	0.92
Preterm birth (< 37 weeks)	8 (10.0)	322 (12.0)	0.72
Hypertensive Disorders of Pregnancy	4 (5.0)	187 (7.0)	0.66
Gestational Diabetes Mellitus	3 (3.8)	109 (4.1)	1.00
Postpartum Hemorrhage	22 (27.5)	490 (18.2)	0.04
Placental Abruption	2 (2.5)	17 (0.6)	0.10
Placenta Previa	10 (12.5)	109 (4.1)	< 0.01

Data are presented as mean ± standard deviation or *n* (%). Postpartum Hemorrhage is defined as greater than 800 mL of estimated blood loss including amniotic fluid in a vaginal delivery or greater than 1500 mL of estimated blood loss including amniotic fluid in a cesarean section

Neonatal outcomes are shown in Table 3. All neonatal outcomes such as birth weight, height, SGA, Apgar score < 7 at 1 and 5 min, umbilical artery pH < 7.1, and NICU admission rates were similar between the two groups.

**Table 3:** Neonatal Outcomes in the Endometriosis and Control groups

Neonatal outcomes	Endometriosis (*N* = 80)	Control (*N* = 2689)	*p*-value
Birth weight (g)	2851 ± 515	2899 ± 560	0.33
Low birth weight (< 2500 g)	13 (16.2)	352 (13.1)	0.51
Height (cm)	49.2 ± 3.3	49.1 ± 3.5	0.72
SGA	2 (2.5)	98 (3.6)	1.00
Male	45 (56.2)	1400 (52.1)	0.53
Apgar score at 1 min < 7	3 (3.8)	210 (7.8)	0.28
Apgar score at 5 min < 7	1 (1.2)	84 (3.1)	0.52
Umbilical artery pH < 7.1	2 (2.5)	26 (1.0)	0.19
NICU admission	17 (21.2)	521 (19.4)	0.78

Data are presented as mean ± standard deviation or *n* (%). *SGA* Small for Gestational Age, *NICU* Neonatal Intensive Care Unit

Univariate and multivariate analyses were performed to identify the independent risk factors for placenta previa. The multivariate analysis was adjusted for endometriosis, pre-pregnancy BMI ≥25 kg/m^2^, maternal age ≥ 35 years old, ART, and multipara (Table 4). Endometriosis was identified as an independent risk factor for placenta previa (aOR, 3.19; 95% CI, 1.56–6.50, *p* < 0.01). ART was also considered to be an independent risk factor for placenta previa (aOR, 2.71; 95% CI, 1.70–4.31, *p* < 0.01).

**Table 4:** Univariate and Multivariate Analysis of Risk Factors associated with Placenta Previa

Factors	Crude OR (95% CI)	Multi-adjusted OR (95% CI)
Endometriosis	3.38 (1.70–6.74)	3.19 (1.56–6.50)
Pre-pregnancy BMI ≥25 (kg/m^2^)	0.62 (0.31–1.23)	0.65 (0.32–1.30)
Maternal age at delivery ≥35 (years)	1.18 (0.82–1.71)	0.92 (0.62–1.36)
ART	2.55 (1.67–3.89)	2.71 (1.70–4.31)
Multipara	1.16 (0.80–1.67)	1.46 (0.99–2.15)

The multivariate analysis was adjusted for each confounding factor including endometriosis, pre-pregnancy BMI ≥25, maternal age at delivery ≥35, ART and multipara by itself. *BMI* Body Mass Index, *ART* Assisted Reproductive Technology, *OR* Odds Ratio, *CI* Confidence Interval

To confirm whether endometriosis was an independent risk factor for PPH, an additional multivariate analysis was performed (Additional file 1: Table S1). We found that endometriosis was not an independent risk factor for PPH after adjusting for confounding factors including pre-pregnancy BMI ≥25 kg/m^2^, maternal age ≥ 35 years old, ART, primipara, placenta previa, and macrosomia (> 4000 g) (aOR, 1.14; 95% CI 0.66–1.98, *p* = 0.64).

An additional analysis was performed to identify patients with a high risk for placenta previa among the endometriosis group (Table 5). We obtained detailed information on pre-pregnancy treatments such as surgeries associated with endometriosis (cystectomy for ovarian endometriosis, ablation or excision of endometrial implants, salpingo-oophorectomy, adhesiolysis, and intestinal resection) and hormone therapies (oral contraceptives, progestogens, and GnRHa). The patients in the surgical treatment group showed a higher risk for placenta previa (crude odds ratio [OR], 4.62; 95% CI, 2.11–10.10, *p* < 0.01) than the non-surgical treatment group. Particularly, patients with a gap of greater than 5 years between pregnancy and surgical treatment showed higher OR for placenta previa (crude OR, 5.92; 95% CI, 1.65–21.30, *p* < 0.01). On the contrary, patients in the non-surgical treatment group were not associated with an increased risk for placenta previa (crude OR, 1.63; 95% CI, 0.19–6.59, *p* = 0.36) (Table 5).

**Table 5:** Univariate Analysis of Pre-pregnancy Treatments associated with Placenta Previa in the Endometriosis group

Pre-pregnancy treatments	With placenta previa (*N* = 10)	Without placenta previa (*N* = 70)	Crude OR (95% CI)
Surgical treatment group	8	41	4.62 (2.11–10.10)
Passed over 5 years at Pregnancy	3	12	5.92 (1.65–21.30)
Non-surgical treatment group	2	29	1.63 (0.19–6.59)
Total	10	70	3.38 (1.70–6.74)

Crude OR was calculated using the control group as a reference. Among 31 patients in the non-surgical treatment group, 26 patients were diagnosed before pregnancy by ultrasound or MRI with ovarian endometriosis (*n* = 22), and symptoms (*n* = 4), and 5 patients were diagnosed with ovarian endometriosis in the 1st trimester of pregnancy by ultrasound. *OR* Odds Ratio, *CI* Confidence Interval

In patients with endometriosis, there was no significant difference in maternal and neonatal outcomes between the surgical treatment group and the non-surgical treatment group (Additional file 1: Tables S2 and S3).

## Discussion

This study aimed to investigate the effects of endometriosis on maternal and neonatal outcomes and to demonstrate whether pre-pregnancy treatments, including surgical treatment and hormone therapies for infertility, ovarian endometriosis, and chronic pain due to endometriosis, affect perinatal outcomes. The main finding of our study was that endometriosis was an independent risk factor for placenta previa after the adjustment of several confounding factors such as fertility treatment, pre-pregnancy BMI, maternal age, and parity. In patients with endometriosis, we found that patients with a history of surgical treatment before pregnancy were associated with an increased risk of placenta previa; on the contrary, patients without pre-pregnancy surgical treatment such as patients who only received hormone therapies or patients coincidentally diagnosed with ovarian endometriosis in the first trimester of pregnancy were not associated with an increased risk.

Our findings are consistent with that of the previous studies that found an association between endometriosis and placenta previa [22–24]. Compared to the recent systematic reviews [17, 25, 26], endometriosis did not increase the risks of preterm birth, HDP, PPH, placental abruption, and SGA in this study. This discrepancy might have been caused by a difference in sample size, patients’ backgrounds, and study designs. Because our hospital specializes in high-risk pregnancies, in the present study, women in the control group may have higher risks for these complications compared to the control groups of the previous studies [12, 13, 15], indicating that it might have been underpowered to show significant difference in maternal and neonatal outcomes.

The underlying mechanisms associating endometriosis to pregnancy complications remain largely unclear. However, several published papers have suggested that chronic inflammation (e.g., cyclooxygenase-2, interleukin-8, prostaglandin E2), adhesions, progesterone-resistant endometrium, and vascularized environment due to endometriosis could lead to various complications during pregnancy [16, 27, 28]. With regard to placenta previa, it has been hypothesized that hyperperistalsis of the uterus may be implicated in abnormal blastocyst implantation [29, 30], and dense pelvic adhesions may inhibit the migration of the placenta away from the internal ostium of uterus. Vercellini et al. reported that a higher incidence of placenta previa was observed in patients with rectovaginal lesions than in patients with peritoneal or ovarian lesions [30]. Additionally, severe cases such as deep infiltrating endometriosis (DIE) and revised American Society for Reproductive Medicine (rASRM) stage IV were significantly associated with a higher risk of placenta previa in subsequent pregnancies [31, 32].

Presently, data on the efficacy of endometriosis treatments such as surgical treatment and hormone therapies on perinatal outcomes in subsequent pregnancies are limited and unclear. To the best of our knowledge, only a few studies have demonstrated an association between pre-pregnancy surgery for endometriosis and the risk of placenta previa [12, 31]. Berlac et al. showed that gynecological surgery for endometriosis before pregnancy had an increased risk for placenta previa in a singleton pregnancy using a national cohort in Denmark (endometriosis, 2.2%; endometriosis with surgery, 3.4%; no endometriosis, 0.4%) [12]. Nirgianakis et al. demonstrated that patients with a history of surgical treatment for DIE presented a higher risk for placenta previa in subsequent pregnancies (endometriosis with surgery, 6.5%; no endometriosis, 0%) [31]. Although the exact reason why the risk for placenta previa increased despite a previous surgery for endometriosis remains unclear, it is possible that patients previously operated on before pregnancy might have exhibited severe endometriosis such as DIE, rASRM stage IV, and uncontrolled pain due to adhesion or ruptured ovarian endometriosis. Consistent with these previous reports [12, 31], patients with a previous surgery in this study were likely to have higher score of rASRM based on limited patients’ data (data not shown). Previous report demonstrated that the recurrence rate of endometriosis after surgery is relatively high, estimated to be 21.5% at 2 years and 40–50% at 5 years [11], and was associated with the duration of follow-up after surgery and the rASRM stage of endometriosis at surgery [33]. In the present study, patients who had a gap of more than 5 years between an surgical treatment and pregnancy had a higher risk of placenta previa. Therefore, increased risk of placenta previa in patients with endometriosis with pre-pregnancy surgical treatment might be due to the higher rate of severe stage of endometriosis or a recurrence of endometriosis. Another possible explanation is that additional adhesions by operative manipulation might have some pathological role in a placenta previa. Additionally, the majority of patients with endometriosis in the non-surgical treatment group were treated conservatively; therefore, they may have had a milder variety of endometriosis compared to patients in the surgical treatment group.

Several limitations of this study should be acknowledged. First, the diagnosis of endometriosis in the non-surgical treatment group was based on ultrasound, MRI, and presence of symptoms, which are less reliable methods than laparoscopy (gold standard). Specifically, symptom-based diagnosis is associated with high risk of misclassification bias and can introduce bias in results especially with a small sample size of 80 patients. Second, the sample size of this single-center retrospective study was smaller than the previous studies; therefore, further studies are required to test our findings in different populations (e.g., races, and regions) [12, 15]. Third, we could not collect sufficient data on the rASRM stage or the presence of DIE for patients with a history of surgical treatment because some patients were operated on at another hospital. Thus, we could not identify the association between placenta previa, pre-pregnancy treatments, and rASRM stage of the patients. Finally, the increased risk of placenta previa in the surgical treatment group may be partly due to the severity of endometriosis rather than surgical treatment itself. However, to determine the effect of surgery itself on pregnancy complications associated with endometriosis, a further study comparing another control group of women who have not been diagnosed with endometriosis but have had an abdominal surgery (e.g., appendectomy) is required.

## Conclusions

Endometriosis is an independent risk factor for developing placenta previa. Additionally, patients with a history of surgical treatment for endometriosis before pregnancy, especially patients with a gap of greater than 5 years between pregnancy and a previous surgery, were associated with increased risk of placenta previa. This may be due to the more severe stage of endometriosis found in these patients. On the contrary, patients who had received only hormone therapies or who were coincidentally diagnosed with ovarian endometriosis in the first trimester of pregnancy were not found to be at greater risk. Thus, patients with a history of surgery require particular attention for placenta previa during pregnancy. Hence, further studies are required to investigate the association between the site and stage of endometriosis or the nature of surgical treatment and placenta previa in a subsequent pregnancy.

## Supplementary information

**Additional file 1: Table S1.** Univariate and Multivariate Analysis of Risk Factors associated with Postpartum Hemorrhage. **Table S2.** Maternal Outcomes in the Surgical treatment and Non-surgical treatment groups. **Table S3.** Neonatal Outcomes in the Surgical treatment and Non-surgical treatment groups.

## Data Availability

The datasets used and/or analyzed during the current study are available from the corresponding author on reasonable request.

## Abbreviations

ART: Assisted reproductive technology
BMI: Body mass index
CI: Confidence interval
DIE: Deep infiltrating endometriosis
DM: Diabetes mellitus
GDM: Gestational diabetes mellitus
GnRHa: Gonadotropin-releasing hormone agonist
HDP: Hypertensive disorders of pregnancy
HT: Hypertension
NICU: Neonatal intensive care unit
OR: Odds ratio
PPH: Postpartum hemorrhage
rASRM: Revised American Society for Reproductive Medicine
SGA: Small for gestational age
