# Supplementary material for: Adverse effects of endometriosis on pregnancy: a case-control study
Source: BMC Pregnancy Childbirth. 2019 Oct 22;19:373. doi: 10.1186/s12884-019-2514-1 (PMC6805464; doi:10.1186/s12884-019-2514-1)
Supplement: Supplementary file 1 — Additional file 1: Table S1. Univariate and Multivariate Analysis of Risk Factors associated with Postpartum Hemorrhage. Table S2. Maternal Outcomes in the Surgical treatment and Non-surgical treatment groups. Table S3. Neonatal Outcomes in the Surgical treatment and Non-surgical treatment groups. [file 12884_2019_2514_MOESM1_ESM.docx]

Supplementary Table 1. Univariate and Multivariate Analysis of Risk Factors associated with Postpartum Hemorrhage.

| Factors | Crude OR | Multi-adjusted OR |
| --- | --- | --- |
|  | (95% CI) | (95% CI) |
| Endometriosis | 1.70 (1.03–2.81) | 1.14 (0.66–1.98) |
| Pre-pregnancy BMI ≥25 (kg/m^2^) | 1.17 (0.88–1.57) | 1.20 (0.89–1.62) |
| Maternal age at delivery ≥35 (years) | 1.17 (0.96–1.42) | 1.02 (0.82–1.26) |
| ART | 2.50 (1.96–3.19) | 2.09 (1.59–2.74) |
| Primipara | 1.62 (1.33–1.98) | 1.55 (1.26–1.92) |
| Placenta previa | 6.15 (4.23–8.96) | 6.08 (4.13–8.95) |
| Macrosomia (>4,000 g) | 3.08 (1.42–6.67) | 3.51 (1.59–7.74) |

The Multivariate Analysis was adjusted for each confounding factor including endometriosis, pre-pregnancy BMI ≥25, maternal age at delivery ≥35, ART, primipara, placenta previa and macrosomia (>4,000 g) by itself. OR: Odds Ratio, CI: Confidence Interval, BMI: Body Mass Index, ART: assisted reproductive technology.

Supplementary Table 2. Maternal Outcomes in the Surgical treatment and Non-surgical treatment groups.

| Maternal outcomes | Surgical treatment | Non-surgical treatment | p-value |
| --- | --- | --- | --- |
|  | (N=49) | (N=31) |  |
| Gestational age (weeks) | 38.2 ± 1.9 | 38.5 ± 2.5 | 0.09 |
| Delivery mode |  |  |  |
| Normal Vaginal | 14 (28.6) | 16 (51.6) |  |
| Instrumental | 5 (10.2) | 2 (6.5) |  |
| Scheduled Cesarean Section | 20 (40.8) | 10 (32.3) |  |
| Emergency Cesarean Section | 10 (20.4) | 3 (9.7) | 0.21 |
| Blood Loss (mL) |  |  |  |
| Normal Vaginal | 595 ± 250 | 889 ± 905 | 0.45 |
| Instrumental | 996 ± 477 | 1,011 ± 392 | 0.97 |
| Scheduled Cesarean Section | 1,421 ± 714 | 1,197 ± 595 | 0.40 |
| Emergency Cesarean Section | 846 ± 335 | 870 ± 288 | 0.91 |
| Preterm birth (<37 weeks) | 4 (8.2) | 4 (12.9) | 0.70 |
| Hypertensive Disorders of Pregnancy | 4 (8.2) | 0 (0.0) | 0.15 |
| Gestational Diabetes Mellitus | 3 (6.1) | 0 (0.0) | 0.28 |
| Postpartum Hemorrhage | 13 (26.5) | 9 (29.0) | 0.80 |
| Placental Abruption | 2 (4.1) | 0 (0.0) | 0.52 |
| Placenta Previa | 8 (16.3) | 2 (6.5) | 0.30 |

Data are presented as mean ± standard deviation or n (%). Postpartum Hemorrhage is defined as greater than 800 mL of estimated blood loss including amniotic fluid in a vaginal delivery or greater than 1,500 mL of estimated blood loss including amniotic fluid in a cesarean section.

Supplementary table 3. Neonatal Outcomes in the Surgical treatment and Non-surgical treatment groups.

| Neonatal outcomes | Surgical treatment | Non-surgical treatment | p-value |
| --- | --- | --- | --- |
|  | (N=49) | (N=31) |  |
| Birth weight (g) | 2,843 ± 445 | 2,865 ± 619 | 0.56 |
| Low birth weight (<2,500 g) | 10 (20.4) | 3 (9.7) | 0.35 |
| Height (cm) | 49.1 ± 2.7 | 49.2 ± 4.1 | 0.35 |
| SGA | 1 (2.0) | 1 (3.2) | 1.00 |
| Male | 30 (61.2) | 15 (48.4) | 0.37 |
| Apgar score at 1 min <7 | 1 (2.0) | 2 (6.5) | 0.56 |
| Apgar score at 5 min <7 | 0 (0) | 1 (3.2) | 0.39 |
| Umbilical artery pH <7.1 | 1 (2.0) | 1 (3.2) | 1.00 |
| NICU admission | 12 (24.5) | 5 (16.1) | 0.42 |

Data are presented as mean ± standard deviation or n (%). SGA: Small for Gestational Age, NICU: Neonatal Intensive Care Unit.
